# Gut Hormones and Inflammatory Bowel Disease

**DOI:** 10.3390/biom15071013

**Published:** 2025-07-14

**Authors:** Jonathan Weng, Chunmin C. Lo

**Affiliations:** 1 Department of Medicine, Tufts Medical Center, Boston, MA 02111, USA; Jonathan.Weng@tuftsmedicine.org; 2 Department of Biomedical Sciences and Diabetes Institute, Heritage College of Osteopathic Medicine, Ohio University, Athens, OH 45701, USA

**Keywords:** glucagon-like peptide-1, glucagon-like peptide-2, glucose-dependent insulinotropic polypeptide, peptide YY, cholecystokinin, apolipoprotein A4, obesity, inflammatory bowel disease

## Abstract

Obesity-driven inflammation disrupts gut barrier integrity and promotes inflammatory bowel disease (IBD). Emerging evidence highlights gut hormones—including glucagon-like peptide-1 (GLP-1), glucagon-like peptide-2 (GLP-2), glucose-dependent insulinotropic polypeptide (GIP), peptide YY (PYY), cholecystokinin (CCK), and apolipoprotein A4 (APOA4)—as key regulators of metabolism and mucosal immunity. This review outlines known mechanisms and explores therapeutic prospects in IBD. GLP-1 improves glycemic control, induces weight loss, and preserves intestinal barrier function, while GLP-2 enhances epithelial repair and reduces pro-inflammatory cytokine expression in animal models of colitis. GIP facilitates lipid clearance, enhances insulin sensitivity, and limits systemic inflammation. PYY and CCK slow gastric emptying, suppress appetite, and attenuate colonic inflammation via neural pathways. APOA4 regulates lipid transport, increases energy expenditure, and exerts antioxidant and anti-inflammatory effects that alleviate experimental colitis. Synergistic interactions—such as GLP-1/PYY co-administration, PYY-stimulated APOA4 production, and APOA4-enhanced CCK activity—suggest that multi-hormone combinations may offer amplified therapeutic benefits. While preclinical data are promising, clinical evidence supporting gut hormone therapies in IBD remains limited. Dual GIP/GLP-1 receptor agonists improve metabolic and inflammatory parameters, but in clinical use, they are associated with gastrointestinal side effects that warrant further investigation. Future research should evaluate combination therapies in preclinical IBD models, elucidate shared neural and receptor-mediated pathways, and define optimal strategies for applying gut hormone synergy in human IBD. These efforts may uncover safer, metabolically tailored treatments for IBD, particularly in patients with coexisting obesity or metabolic dysfunction.

## 1. Obesity, Inflammation, and Inflammatory Bowel Disease

Obesity has become a global epidemic, affecting more than 40% of adults in the United States [1]. Inflammatory bowel disease (IBD), including Crohn’s disease (CD) and ulcerative colitis (UC), was once primarily linked to weight loss and malnutrition [2,3]. However, it is now common for patients with IBD to also be obese (body mass index [BMI] ≥ 30 kg/m^2^), with recent studies showing that 15% to 40% are obese and an additional 20% to 40% are overweight (25 kg/m^2^ ≤ BMI < 30 kg/m^2^), mirroring trends in the general population [4,5,6]. Obesity-induced low-grade inflammation and immune dysfunction are linked to many chronic metabolic diseases, including IBD [6,7,8,9,10]. Excess caloric intake, particularly through a high-fat diet (HFD), induces local inflammation in various organs—such as the small intestine, adipose tissue, liver, and skeletal muscle—which play critical roles in maintaining energy homeostasis [11,12,13]. This inflammatory milieu contributes to the development of IBD [6,10,14].

Gut microbiota, such as Bacteroidetes and Firmicutes, metabolize indigestible dietary fiber into short-chain fatty acids (SCFAs) and bile acid derivatives in the colon [15]. SCFAs serve as a major energy source for colonic epithelial cells, regulate metabolism, and exert immunomodulatory effects that help maintain a balance between pro- and anti-inflammatory states [16]. HFDs disrupt gut homeostasis by altering the microbiota composition—such as by increasing the Firmicutes/Bacteroidetes ratio—reducing SCFA production, and increasing endotoxemia, which leads to intestinal dysbiosis, compromised epithelial barrier function, and enhanced intestinal permeability [16,17].

HFD-induced obesity increases the mass of mesenteric adipose tissue around the small intestine [18] and enlarges adipocytes, which produce chemokines and chemotactic adipokines [19]. This process promotes the production of inflammatory cytokines in both mesenteric and visceral adipose tissue [14,20], leading to systemic inflammation [21,22] and heightened intestinal cytokine production [14,23,24]. An HFD also enhances macrophage infiltration [25], disrupts the mucosal barrier, and permits luminal microbiota to incite sustained inflammatory responses in the mucosa and submucosa of the small intestine and colon [25,26,27]. Increased intestinal permeability due to mucosal inflammation allows bacteria or bacterial products, such as lipopolysaccharide, to cross the intestinal barrier [17,28,29] and enter the systemic circulation, thereby triggering inflammation in peripheral tissues and insulin resistance [30,31,32].

Clinical studies have linked diets high in saturated, monounsaturated, and polyunsaturated fatty acids (FAs) to increased risk of CD and UC [10,23]. In patients with CD, hypertrophied mesenteric fat—also known as creeping fat—is commonly observed [33,34,35,36], and elevated levels of adiponectin and cytokines in mesenteric fat further exacerbate mucosal inflammation [37]. IBD patients exhibit excessive recruitment and activation of immune cells across multiple cell subsets, including myeloid cells in the lamina propria [38,39,40,41], natural killer cells in the mucosa [42], activated mononuclear cells [43,44], and mucosal T cells [45,46,47,48]. Furthermore, dysregulation of innate lymphoid cells, which normally maintain mucosal immunity through IL-22-mediated induction of antimicrobial peptides, can contribute to the development and perpetuation of IBD-associated inflammation [49].

**Figure 1:**
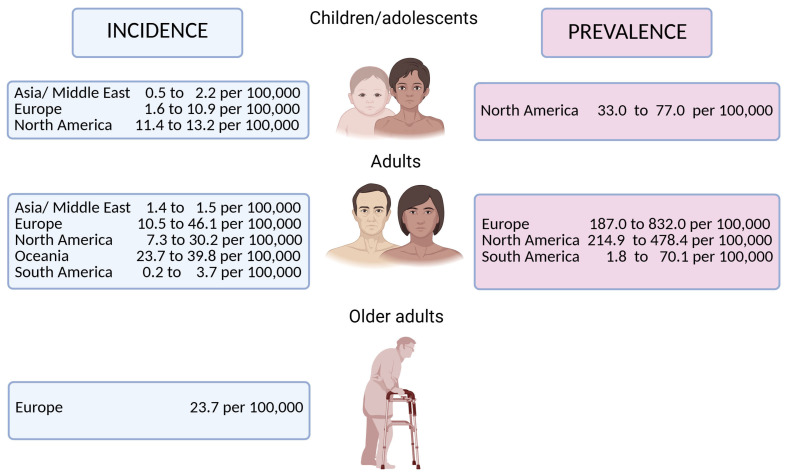
Epidemiology of inflammatory bowel disease across age groups. Data adapted with permission from Caron et al. [50].

Epidemiological data (Figure 1) indicate that the annual incidence of adult IBD is higher in Europe and North America than in Asia, the Middle East, or South America [50]. Prevalence estimates range from 187 to 832 per 100,000 in Europe, 215 to 478 per 100,000 in North America, and 2 to 70 per 100,000 in South America [50]. Obesity has been associated with an increased risk of CD, further implicating metabolic dysfunction in IBD pathogenesis in Table 1 [51,52].

Patients with IBD often face both intestinal and systemic complications, including intestinal perforations, toxic megacolon, abscesses, and an increased risk of colon cancer [53,54]. The pathogenesis of IBD involves mucosal injury, characterized by a compromised mucin layer and disrupted tight junctions, along with an inflammatory response triggered by luminal microbes that penetrate the lamina propria and by dysregulated CD4^+^ T lymphocytes secreting pro-inflammatory cytokines [26,55,56,57,58]. Therapeutic approaches for IBD focus on reducing local and systemic inflammation to mitigate acute flares; sustain clinical, endoscopic, radiologic, and histologic remission; and address intra- or extra-intestinal complications. Treatment options include aminosalicylates, glucocorticoids, immunomodulators (e.g., thiopurines, methotrexate, cyclosporine), biologic agents (e.g., tumor necrosis factor inhibitors, anti-interleukin antibodies, anti-integrin antibodies), and small-molecule inhibitors (e.g., Janus kinase inhibitors, sphingosine-1-phosphate receptor modulators) [2,59,60,61,62]. However, patients taking these medications are susceptible to a broad spectrum of adverse effects, ranging from mild symptoms, such as nausea, vomiting, and fatigue, to more serious complications like infection and malignancy [63,64,65].

There is, therefore, a pressing need for novel therapies that are both effective and better tolerated. Several gut-derived hormones—secreted before, during, or after meals—regulate food intake and energy homeostasis [66,67,68,69]. These hormones, which include both enteroendocrine hormones (such as glucagon-like peptide-1, glucose-dependent insulinotropic polypeptide, peptide YY, and cholecystokinin) and enterocyte-derived hormones (such as apolipoprotein A4), respond to dietary nutrients and play critical roles in energy homeostasis through regulation of lipid and glucose metabolism across multiple organs [67,69,70,71,72,73,74]. Emerging evidence suggests that these hormones may attenuate the development of CD and UC [75,76,77]. Although the precise mechanisms remain elusive, their roles in improving metabolic homeostasis and reducing systemic inflammation offer a promising therapeutic avenue for IBD.

## 2. Glucagon-like Peptide

Glucagon-like peptide-1 (GLP-1) is a post-translational product of the proglucagon protein, encoded by the GCG gene and processed by the enzyme prohormone convertase 1/3 [78]. Dietary lipids, glucose, or mixed meals stimulate GLP-1 production by enteroendocrine L-cells in the small intestine [78,79,80,81,82] where its sister molecule, glucagon-like peptide-2 (GLP-2), is also synthesized and secreted [83,84]. The presence of the GLP-1R in the vicinity of these cells is critical for its physiological actions [79,85]. The early phase of lipid-mediated GLP-1 secretion in the lymph requires chylomicron formation [82]. GLP-1 then traverses the lamina propria, entering the lymphatic system [80,82] or capillaries where it is degraded by dipeptidyl peptidase-4 (DPP-4) expressed on endothelial membranes [86]. DPP-4 cleaves GLP-1 more rapidly than GLP-2, giving GLP-1 a short in vivo half-life of 1–2 min, compared to 7 min for GLP-2 in humans [84]. Because DPP-4 activity is higher in plasma than in lymph, GLP-1 concentrations are significantly higher in intestinal lymph than in venous plasma [87].

In obesity, hepatocyte-derived DPP-4 activates inflammatory pathways in adipose tissue macrophages via the caveolin-1 and protease-activated receptor 2 pathways, leading to the activation of extracellular signal-regulated kinases 1 and 2 and nuclear factor kappa B (NF-κB) signaling [88] and promoting the release of pro-inflammatory mediators including cytokines, chemokines, and neuropeptides [89]. In contrast, DPP-4 inhibition reduces inflammation and oxidative stress [90,91]. DPP-4 inhibitors, such as anagliptin [92,93], sitagliptin [94], vildagliptin [95], and linagliptin [96], enhance endogenous GLP-1 activity by preventing its degradation by DPP-4, leading to improved insulin secretion, reduced fasting plasma glucose, lower hemoglobin A1c, and improved glucose homeostasis [97,98]. In mouse models of experimental colitis, DPP-4 inhibition increases GLP-2 levels, attenuates inflammation, reduces disease severity, and supports mucosal healing through the suppression of T cell proliferation and cytokine production [75,99,100,101,102]. However, clinical studies of DPP-4 inhibitors in IBD have yielded mixed results. A primary random-effect meta-analysis of patients receiving DPP-4 inhibitors for 52 weeks to 5 years found no increased risk of developing IBD [103]. Furthermore, patients treated with DPP-4 inhibitor/metformin combination therapy had a lower risk of autoimmune disease, including IBD, compared to those receiving non-DPP-4 inhibitor/metformin regimens [104]. Some studies report an inverse correlation between IBD activity and serum DPP-4 levels, while others suggest that long-term DPP-4 inhibitor use in patients with type 2 diabetes may increase IBD risk [105,106]. DPP-4 inhibitors are widely used in clinical practice as oral antidiabetic agents and may also improve gut barrier function via GLP-2-dependent mechanisms in murine obesity models [107]. Further investigation is needed to evaluate the therapeutic potential of GLP-1/GLP-2 receptor agonists and DPP-4 inhibitors—alone or in combination—for the treatment of IBD.

Functioning as both an anorexigenic neuropeptide and incretin hormone [97,108,109,110,111,112,113], GLP-1 interacts with GLP-1R on vagal afferent neurons [78,80,114,115,116], transmitting satiety signals to the nucleus of the solitary tract and the hypothalamus [108,109,110,111,112]. This interaction also enhances insulin secretion and sensitivity, contributing to postprandial and fasting glucose regulation [97,113,117,118]. However, the therapeutic utility of native GLP-1 is limited due to its rapid degradation by DPP-4. Therefore, synthetic GLP-1R agonists, such as dulaglutide, liraglutide, and semaglutide, have been developed. These agents not only modulate glucose homeostasis via pancreatic α and β cells but also act on the central nervous system to suppress appetite [119] and enhance insulin sensitivity [120]. In addition to their metabolic effects, GLP-1 and GLP-1R agonists slow gastric emptying [121,122], reduce triglyceride (TG) absorption without altering pancreatic lipase activity, and attenuate intestinal production of TGs and cholesterol associated with VLDL/chylomicron synthesis [123,124,125]. They also lower fasting and postprandial TG levels by inhibiting insulin-mediated lipolysis [117,118,126] and promoting FA uptake in adipose tissues expressing GLP-1R [127,128], reduce VLDL-TG production and hepatic lipid accumulation [129,130], and attenuate fat mass and body weight gain in both mice and human subjects [118,129,131,132]. A meta-analysis of overweight and obese patients with or without diabetes demonstrated that GLP-1R agonists improve plasma lipids and glycemic control and induce weight loss [133], leading to their widespread use in treating type 2 diabetes and obesity [108,134,135,136].

GLP-1 also exerts anti-inflammatory, antioxidative, and anti-apoptotic effects [65,137,138]. In obese mice, GLP-1 and its receptor agonists reduce macrophage population and lower the expression of pro-inflammatory cytokines such as interleukin-6 (IL-6), interleukin-1β (IL-1β), and tumor necrosis factor-α (TNF-α) in adipose tissue of animals and human subjects [138,139,140]. Abundant GLP-1 expression in intestinal intraepithelial lymphocytes of animals—which serve as both a barrier and a repair mechanism in the small intestine—suggests an important role in pathogen clearance and epithelial protection [141,142,143]. GLP-1R agonists have been shown to reduce cytokine production by acting on intraepithelial lymphocyte GLP-1Rs, elevating immunomodulatory and antimicrobial factors in the small intestine of mice with UC [141,144]. These agents also increase cyclic adenosine monophosphate (cAMP) levels and modulate pro-inflammatory genes in intraepithelial lymphocytes [141], contributing to decreased intestinal inflammation in animals [145,146]. GLP-1R agonists also upregulate barrier-protective genes and attenuate multiple colonic cytokines, including TNF-α, interleukin-1α (IL-1α), T cell activation gene-3, stromal cell-derived factor-1, and macrophage colony-stimulating factor [147,148]. In mouse models of colitis, the administration of GLP-1 has been reported to alleviate colonic inflammation and colon damage by reducing the expression of pro-inflammatory cytokine IL-1β, increasing goblet cell numbers, preserving intestinal epithelial architecture, and expanding intestinal crypts [85,149,150]. Thus, GLP-1R agonists may limit IBD progression through both direct enhancement of immune defense and intestinal barrier function and indirect effects related to improved metabolic homeostasis and reduced systemic inflammation.

GLP-1 signaling also affects intestinal motility. In healthy humans, the GLP-1R antagonist exendin 9–39 stimulates duodenal motility in response to intestinal nutrients [151]. In contrast, native GLP-1 or GLP-1R agonists inhibit small intestinal motility in healthy subjects and patients with type 2 diabetes or irritable bowel syndrome [152,153,154,155,156]. Rodent models with vagal afferent denervation or knockdown confirm that this inhibitory effect is mediated via vagal afferents [157,158,159]. Intestinal motility in IBD is variable—some patients experience reduced motility and constipation, while others have increased motility and diarrhea [160,161]. MRI studies have shown reduced motility indices in the ileum of patients with small bowel CD [161,162], with the degree of motility impairment correlating with inflammatory markers such as C-reactive protein and fecal calprotectin [163,164]. Accordingly, anti-inflammatory therapies, including aminosalicylates, corticosteroids, immunomodulators, and biologics, aim to reduce intestinal inflammation and restore motility [165].

While GLP-1R agonists represent promising therapeutic agents due to their anti-inflammatory and regenerative properties, their potential to further impair intestinal motility and worsen constipation must be considered, particularly in IBD patients prone to slow transit. Over half of users experience gastrointestinal side effects, including nausea, vomiting, and diarrhea, which often lead to treatment discontinuation [166,167,168]. These symptoms can overlap with those of IBD, raising concerns about the risk of more severe complications, such as ileus or bowel obstruction [169,170,171,172]. Reassuringly, recent studies have demonstrated that GLP-1R agonist therapy is not associated with increased risk of serious gastrointestinal adverse events, including ileus, intestinal obstruction, IBD-related hospitalization, corticosteroid use, medication escalation, or IBD-related surgery [131,173,174].

GLP-2, a co-secreted peptide, also exerts multiple physiological effects. It suppresses gastric secretion [175], gastric motility [176], and crypt cell apoptosis [177,178], while stimulating intestinal nutrient transport [179,180,181,182], intestinal blood flow [183,184,185], crypt cell proliferation [186,187], and gut barrier integrity [188,189]. GLP-2 has been identified as a novel intestinal growth factor that promotes the proliferation of crypt cells and mucosal epithelium and suppresses enterocyte apoptosis in mice [187,190,191]. GLP-2 signals via the GLP-2 receptor, localized to the myenteric and submucosal plexuses [192], to induce the release of growth factors from subepithelial myofibroblasts through the phosphoinositide 3-kinase (PI3K)/Akt pathway [193]. GLP-2 or its analogs, such as teduglutide, activate the cAMP/protein kinase A-dependent pathway to promote small intestinal growth [194].

In animal models of colitis, GLP-2 acts via vasoactive intestinal polypeptide (VIP) neurons in the submucosal plexus to reduce pro-inflammatory cytokines, such as TNF-α and interferon-γ (IFN-γ), and mitigate mucosal injury by increasing crypt cell proliferation, upregulating suppressor of cytokine signaling 3 expression, and reducing signal transducer and activator of transcription 3 signaling, crypt cell apoptosis, and IGF-1 production [195,196,197,198,199,200]. Thus, the dual function of GLP-2 as an intestinal growth factor and anti-inflammatory mediator makes it a promising candidate for IBD treatment. In a clinical trial involving 71 patients with CD, teduglutide induced remission in 50% of patients, increased plasma citrulline levels, and had a comparable safety profile to placebo, supporting its potential as a novel therapy for mucosal healing in moderate-to-severe CD [201]. Further clinical studies are needed to explore the immunomodulatory effects of GLP-2.

## 3. Glucose-Dependent Insulinotropic Polypeptide

Glucose-dependent insulinotropic polypeptide (GIP), previously known as gastric inhibitory polypeptide, is secreted by enteroendocrine K cells in the duodenum and jejunum in response to dietary lipids and other nutrients [202,203]. The biologically active form, GIP_1–42_, consists of 42 amino acids and is derived from a 153-amino-acid preprohormone, proGIP, in humans [204] or a 144-amino-acid precursor in rodents, which is secreted in most K cells [205,206]. GIP_1–42_ is generated from proGIP through the removal of the 51 N-terminal residues and 60 C-terminal residues by prohormone convertases 1/3 in enteroendocrine K cells of the proximal small intestine [207,208]. Additionally, a truncated form, GIP_1–30_, is produced from proGIP via the action of prohormone convertase 2, followed by C-terminal amidation by peptidyl-glycine α-amidating monooxygenase [207,208]. Dietary lipids and glucose synergistically stimulate the secretion of GIP into the lymph, with lipids serving as a more potent stimulus than glucose [203].

GIP plays several roles in metabolism and energy homeostasis. Infusion of GIP agonists has been shown to inhibit gastric acid secretion by suppressing gastrin release independently of gastric emptying [209,210,211,212]. At the pancreatic level, GIP enhances insulin secretion [213,214,215,216,217], which helps regulate postprandial blood glucose levels by promoting the disposal of nutrients into adipose tissues [202,218]. Furthermore, GIP reduces diet-induced weight gain by lowering food intake and increasing FA oxidation, thereby improving glucose homeostasis via GIP receptors (GIP-R) [219]. In obesity, downregulated GIP-R expression and impaired downstream signaling disrupt FA and glucose uptake in white adipose tissue (WAT) [220]. In the postprandial period, GIP enhances lipoprotein lipase-mediated clearance of chylomicron-associated TGs [221,222,223] and increases adipose tissue storage by facilitating FA uptake directly via GIP-R [126,218,222,224] or indirectly by augmenting insulin-mediated FA incorporation [225,226]. In the fasting state, GIP promotes lipid excretion [73,227]. In addition, GIP activates brown adipose tissue (BAT) thermogenesis, leading to increased FA beta-oxidation and reduced fat deposition, which in turn mitigates systemic inflammation by lowering cytokine production in WAT [9,34,128,218,228,229,230,231,232,233,234]. Overall, by enhancing insulin sensitivity [202] and reducing HFD-induced macrophage infiltration along with pro-inflammatory chemokine and cytokine production [235], GIP contributes to lower plasma TG levels [128,236,237] and a reduction in systemic inflammation [235].

Elevated plasma GIP levels, impaired enteric neuronal function, and diminished colonic smooth muscle responses have been observed in animal models of colitis [238]. GIP-R is expressed on the basolateral surface of epithelial cells in the duodenum and proximal small intestine [85], as well as on monocytes and macrophages [149]. In mice, the global knockout of GIP-R reduces bone marrow neutrophil counts and inflammation [149]. In bone marrow chimeric models with GIP-R deletion restricted to immune cells, there is a reduction in IL-33 expression and regulatory T cells (CD4^+^CD8^−^CD25^+^FOXP3^+^), along with increased IL-10 levels in F4/80^+^ cells within WAT [239]. Moreover, GIP-R deletion in myeloid cells disrupts type 2 immune cell networks in WAT [239]. These findings suggest a regulatory role for GIP-R signaling in immune homeostasis in WAT.

However, the expression profiles of GIP and GIP-R in the small intestine of healthy individuals, patients with IBD, and animal models of colitis remain incompletely characterized. Further studies are needed to evaluate the effects of GIP or GIP-R agonists on intestinal inflammation and tissue morphology in IBD.

## 4. Peptide YY

Peptide YY (PYY) is a 36-amino-acid hormone produced by enteroendocrine L cells located in the ileum, colon, and rectum [240,241,242]. Its release is primarily triggered by intestinal nutrients, particularly long-chain FAs [243,244,245]. Two main forms of PYY circulate in the bloodstream: PYY_1–36_ and PYY_3–36_. PYY_3–36_ is generated when DPP-4 cleaves tyrosine and proline from the N-terminus of PYY_1–36_ [246]. Given that plasma levels of DPP-4 exceed those in the lymph [87], PYY concentrations are higher in lymph than in plasma [87]. In the fasting state, PYY_1–36_ predominates, whereas PYY_3–36_ is the major circulating form after meals [246]. Both forms of PYY interact with neuropeptide Y (NPY) receptors. PYY_1–36_ binds with similar affinity to NPY receptor type 1 and type 2 (NPY-Y2), while PYY_3–36_ shows a higher selectivity for NPY-Y2 [247,248]. The NPY-Y2 receptor is expressed in intestinal cells, peripheral parasympathetic and sympathetic sensory neurons, and multiple regions of the central nervous system [249]. When nutrients reach the ileum, the release of PYY_3–36_ by intestinal L cells helps slow gastric emptying and intestinal motility by inhibiting gallbladder emptying and suppressing secretion of gastric acid and pancreatic enzymes [250]. PYY_3–36_ also decreases caloric intake and increases energy expenditure in animals [250,251,252] and human subjects [253,254] by reducing gastric emptying [249] or by acting on NPY-Y2 receptors in the hypothalamus after crossing the blood–brain barrier [249].

Obese individuals show an impaired PYY response to HFDs, with reduced plasma PYY levels observed in both obese mice [255] and humans [254,256], in contrast to lean individuals, who display increased PYY levels in response to HFDs [257]. Fasting levels of PYY_3–36_ are inversely related to adiposity in humans [254,258,259]. In mice, PYY deficiency leads to increased subcutaneous and visceral adiposity due to elevated caloric intake [247,257], whereas administering PYY can mitigate this adiposity through a reduction in food intake and body weight in both HFD-induced obese animals and obese human subjects [253,254,257]. PYY also appears to favor fat oxidation as an energy source in obese mice [260,261] and increases whole-body energy expenditure independent of the effects of food intake in mice and human subjects [253,262].

PYY exhibits anti-inflammatory properties in WAT by downregulating pro-inflammatory factors such as NF-κB and IL-6 in obese mice, thereby mitigating the development of metabolic diseases associated with obesity [260,263]. Although PYY levels are negatively correlated with adiposity [247,254,258,259], PYY sensitivity appears preserved in the context of obesity or HFD feeding [247,254,256,258,259], and exogenous PYY attenuates obesity-related inflammation in WAT [260,263]. However, the extent to which PYY mediates anti-inflammatory crosstalk between the small intestine and WAT remains unclear and warrants further investigation.

CD is primarily a T helper 1 (Th1) cell-mediated disorder characterized by macrophage activation; increased production of pro-inflammatory cytokines like IFN-γ, TNF-α, and IL-6; and elevated levels of Th1 cytokines such as IL-2 and IL-12, with only minor alterations in Th2 cytokines like IL-4 and IL-10 [264,265]. Intestinal macrophages, located in the lamina propria, submucosa plexus, and muscularis externa, regulate both motility and mucosal inflammation [266]. In murine macrophages, PYY enhances immune response by promoting adhesion, chemotaxis, phagocytosis, and superoxide anion production [267,268]. However, PYY_3–36_ also suppresses the secretion of pro-inflammatory cytokines TNF-α and IL-6 from lipopolysaccharide-stimulated macrophages in vitro [265].

Reduced PYY levels have been observed in the colonic tissues of patients with IBD [269,270]. In mice with colitis, PYY_3–36_ alleviates colonic inflammation by reducing myeloperoxidase activity and lowering both colonic and systemic levels of TNF-α and IL-6. It also decreases the percentage of IFN-γ-producing CD4^+^ T cells in the spleen and the proportion of Th1/Th2 splenocytes, reducing colon tissue damage, weight loss, and mortality [265]. PYY also interacts with the Y1 receptor on intestinal epithelial cells to promote epithelial proliferation via mitogen-activated protein kinase signaling pathways [271,272].

Taken together, these findings suggest that PYY is a potential candidate for attenuating IBD severity. However, whether exogenous PYY administration can similarly reduce intestinal inflammation in animal models or patients with IBD remains to be fully explored.

## 5. Cholecystokinin

Cholecystokinin (CCK) is synthesized and secreted by enteroendocrine I cells in the mucosal epithelium of the duodenum and proximal jejunum in response to luminal nutrients [273,274,275,276]. As both a gut hormone and neuropeptide within the enteric nervous system [275,277,278,279], peripheral CCK reduces meal size by delaying gastric emptying [273,280] and facilitates digestion by increasing intestinal motility [275,277,278,279,281], stimulating gallbladder contraction, and enhancing pancreatic exocrine secretion [273,274,282,283]. Centrally, CCK inhibits food intake [284], triggers pain responses [285], facilitates memory performance [286], and attenuates anxiety [287].

CCK mediates its physiological effects in peripheral tissues and the central nervous system through two main receptors, the CCK_1_ receptor (CCK-1R) and the CCK_2_ receptor (CCK-2R) [282,283,288,289]. CCK-1R is highly expressed in the small intestine, pancreas, vagus nerve, nucleus tractus solitarius, and hypothalamus [289,290], whereas CCK-2R is present in the hypothalamus, vagus nerve, and gastric mucosa [273,288,291]. Studies in knockout (KO) mice reveal distinct roles for these receptors: CCK-1R KO mice exhibit normal body weight [292] but altered feeding behavior, consuming larger, less frequent meals, especially when fed a HFD [293]. In contrast, CCK-2R KO mice display increased food intake, elevated energy expenditure, and development of obesity [294,295,296], suggesting divergent roles for CCK-1R and CCK-2R in maintaining energy balance. In addition, CCK-1R activation in the pancreas promotes insulin release [297,298,299,300,301,302,303] and improves postprandial glucose control by potentiating glucose-mediated insulin secretion in type 2 diabetes [304,305,306]. Collectively, these actions position CCK as a key regulator of energy homeostasis, lipid and glucose metabolism, and neurobehavioral processes.

Endotoxemia lowers circulating CCK levels, compromises intestinal barrier integrity, and exacerbates inflammation in the ileum and colon [307,308,309], suggesting that inflammation can impair CCK synthesis. In rodent models, CCK administration mitigates colitis [310] by preserving mucosal barrier function and suppressing intestinal and systemic inflammation [309,311]. CCK also protects against gastric and colonic ulceration [312,313,314,315,316,317] by stimulating sensory nerves and increasing local blood flow to the ulcerated area, possibly mediated by nitric oxide [313].

CCK exerts immunomodulatory effects through multiple mechanisms. In human colonic lamina propria, CCK-1R activation inhibits lymphocyte proliferation [318], while in peripheral lymphocytes, it promotes mitogenesis via calcium signaling [319]. In animal models of intestinal inflammation, CCK suppresses Th1 and Th17 differentiation, enhances Th2 cytokine production, and induces regulatory T cells expressing forkhead box protein P3 (FOXP3), through both CCK-1R and CCK-2R [310,320,321,322]. Moreover, in rat pulmonary interstitial macrophages, CCK acts via CCK-1R and CCK-2R to suppress lipopolysaccharide-induced IL-1β production by activating the cAMP-protein kinase A pathway and inhibiting p38 kinase and NF-κB [323].

These findings suggest that CCK exerts anti-inflammatory and immunomodulatory effects in the gastrointestinal tract via neural circuits, calcium signaling in lymphocytes, and cytokine secretion from macrophages. However, it remains to be determined whether its action through CCK-1R, CCK-2R, or both is critical to limiting the development of IBD. Further investigations are needed to explore how CCK signaling through these receptors may inhibit colonic inflammation and IBD development or progression.

## 6. Apolipoprotein A4

Apolipoprotein A4 (APOA4) is synthesized by enterocytes in the jejunum and ileum in response to dietary lipids and is secreted with TG-rich chylomicron particles into the lymph [324,325]. In circulation, APOA4 associates with chylomicron remnants, high-density lipoproteins (HDLs), or exists as lipoprotein-free particles [326,327]. Functioning as a short-term satiating factor, APOA4 reduces meal size via vagal pathways and increases meal frequency without altering total daily food intake [74,325,328,329]. It also regulates lipid transport within chylomicrons [330], facilitates chylomicron clearance [331], and enhances FA uptake by adipose tissue through stimulation of lipoprotein lipase-mediated lipolysis of circulating TG-rich lipoproteins [332]. APOA4 limits HFD-induced weight gain and adiposity by stimulating sympathetic activity, increasing BAT thermogenesis, and boosting hepatic FA oxidation and overall energy expenditure [328,332,333].

Low plasma APOA4 concentrations are linked to coronary artery disease in humans [334,335,336]. Conversely, APOA4 overexpression or recombinant APOA4 administration protects against HFD-induced atherosclerosis by lowering triglycerides, reducing vascular inflammation, and raising HDL cholesterol [337,338,339,340,341,342,343,344]. It also inhibits the development of hepatic steatosis by enhancing liver FA oxidation [333,337,345]. Beyond metabolic effects, APOA4 possesses anti-inflammatory [346,347], antioxidant [348,349], and anti-atherogenic properties [74,338,346,350]. It has also been shown to improve the development of colitis [77] and atherosclerosis [74,338,346,350]. In mice, loss of APOA4 increases pro-inflammatory cytokines in the small intestine [77], adipose tissue [347] and liver [351], while elevated APOA4 levels suppress cytokine production in these tissues [77,347,351] and in atherosclerotic plaques [346] by inhibiting IκB kinase and c-Jun N-terminal kinase (JNK) signaling [347]. APOA4 has been reported to be a potent endogenous inhibitor of lipid oxidation [348,349] and attenuates oxidant-induced apoptosis by modulating intracellular glutathione redox balance in mice [352]. In addition, APOA4 stabilizes adherens junctions in the small intestine [353] and inhibits monocyte activation by lipopolysaccharide [346]. Reduced APOA4 levels have been observed in the small intestine [353,354] and plasma [355] of patients with IBD, whereas acute administration of APOA4 alleviates inflammatory symptoms in animals with experimental colitis [77]. It remains unclear whether IBD development is primarily improved by APOA4 directly fortifying mucosal integrity and dampening local inflammation, or by indirectly reducing systemic inflammation via enhanced FA oxidation and thermogenesis. Elucidating the key mechanism will clarify its therapeutic potential in IBD.

Low-density lipoprotein receptor-related protein 1 (LRP1) is a 600 kDa multi-ligand transmembrane receptor [356,357] that facilitates FA uptake in adipose tissue [358,359,360] and promotes the catabolism of VLDL and chylomicron remnants in hepatocytes via apolipoprotein E binding [361,362,363,364,365]. LRP1 also protects against atherosclerosis [366,367,368] by inhibiting lipid accumulation and smooth muscle proliferation [369,370,371], enhancing macrophage cholesterol efflux [372,373], and attenuating inflammation through suppression of NF-κB and JNK pathways [357,374,375]. In colonic inflammation, LRP1 expression is elevated in M1 macrophages [376], which clear bacteria and necrotic debris [377,378,379,380]. Macrophage LRP1 further reduces apoptosis through Akt signaling and diminishes inflammation by modulating NF-κB activity to decrease IL-1β, IL-6, and TNF-α expression [374,381]. LRP1 engagement limits intestinal inflammation [382] by recruiting adaptor protein binding to the LRP-1 β-chain [383,384,385], preventing JNK nuclear translocation by binding JNK-interacting proteins [385], and downregulating NF-κB signaling [374,381,386].

While LRP1 serves as a novel APOA4 receptor in adipose tissue for glucose homeostasis [387], it remains to be determined whether APOA4-LRP1 interactions in the intestine can similarly suppress inflammatory cytokine production and restrict the development of colitis or IBD [77].

## 7. Synergistic Actions of Gut Hormones in IBD Regulation

**Figure 2:**
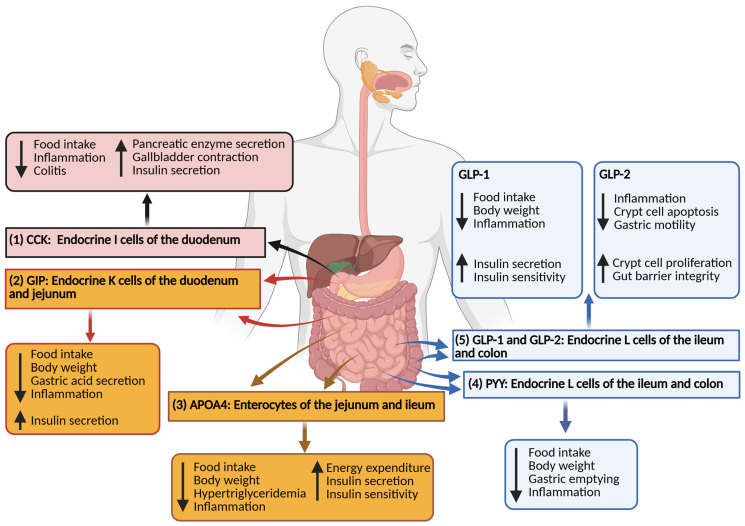
Overview of gut hormones and their physiological roles: (1) CCK, produced by duodenal I cells, induces short-term satiety; stimulates pancreatic enzyme secretion, gallbladder contraction, and insulin secretion; and reduces inflammation and colitis. (2) GIP, secreted by duodenal and jejunal K cells, decreases food intake and body weight; inhibits gastric acid secretion; attenuates inflammation; and enhances insulin secretion. (3) APOA4, released by jejunal and ileal enterocytes, reduces short-term meal size and body weight; lowers hypertriglyceridemia and inflammation; and promotes energy expenditure, insulin secretion, and insulin sensitivity. (4) PYY, produced by ileal and colonic L cells, slows gastric emptying; decreases food intake and body weight gain; and attenuates inflammation. (5) GLP-1, secreted by ileal and colonic L cells, reduces food intake and body weight; enhances insulin secretion and sensitivity; and attenuates inflammation. GLP-2, co-secreted with GLP-1, reduces inflammation and crypt cell apoptosis; suppresses gastric motility; and promotes crypt cell proliferation and gut barrier integrity.

Gut hormones are secreted along the length of the small intestine by specialized enteroendocrine and enterocyte cells: GLP-1 from jejunal L cells, PYY from ileal L cells, GIP from duodenal and jejunal K cells, CCK from duodenal and jejunal I cells, and APOA4 from jejunal and ileal enterocytes (Figure 2) [67,73,202,203,247,324,325,388]. Locally, these hormones help maintain epithelial integrity and modulate mucosal immunity, thereby limiting the development of IBD [73,77,144,202,247,269,310,388,389].

Combination therapies that target multiple gut hormones have shown promise not only for weight loss and metabolic control but also as potential anti-inflammatory strategies in IBD [128,227,390,391,392,393]. Dual GIP/GLP-1R agonists engage both receptors simultaneously [227,391,394], resulting in enhanced FA uptake in adipose tissue and improved glucose disposal in adipose and skeletal muscle [120,395,396]. These actions reduce systemic inflammation and obesity [128,227,391], and GLP-1 alone has been demonstrated to attenuate colonic inflammation in mouse models of UC [141,144].

Tirzepatide, a dual GIP/GLP-1R agonist, has demonstrated efficacy in reducing body weight and limiting progression to type 2 diabetes in patients with obesity and prediabetes [397], suggesting that combined GIP/GLP-1 therapy may offer synergistic benefits in obesity-associated IBD. However, side effects such as nausea, constipation, decreased appetite, dyspepsia, diarrhea, and vomiting have been reported in patients with type 2 diabetes treated with tirzepatide [398]. Further research is needed to clarify whether GIP or GIP-1R agonists can also attenuate intestinal inflammation in IBD [399], and clinical studies further evaluating adverse events associated with GIP/GLP-1 combination therapy are warranted.

Co-administration of GLP-1 and PYY_3–36_ produces additive reductions in food intake in both humans and mice compared to either hormone alone [392,400,401] and enhances insulin sensitivity through restoration of pancreatic beta-cell function and neuronal activation in mice [402]. PYY also stimulates intestinal APOA4 synthesis [403], and APOA4 subsequently enhances both the production and effects of CCK [393,404]. Independently, CCK and APOA4 each confer protection against inflammation and colitis [77,309,310,311,346,347] through neural circuits that regulate energy homeostasis, reinforce epithelial barrier integrity, and suppress inflammatory signaling [276,325,328,332,333,337,404,405,406,407].

However, whether combinations such as GLP-1/PYY, PYY/APOA4, or APOA4/CCK offer superior protection against colonic inflammation and IBD has yet to be explored. Given these interconnections, future studies should examine how gut hormones interact at the receptor level and within neural pathways to regulate mucosal immunity. Preclinical IBD models will be crucial for evaluating multi-hormone regimens, optimizing dosing strategies, and uncovering new therapeutic targets. Illuminating the mechanisms underlying gut hormone synergy could open novel pharmacologic avenues for treating IBD in the context of obesity and other metabolic disorders.

## Figures and Tables

**Table 1:** Baseline characteristics at the time of IBD diagnosis. Data adapted from Sehgal et al. [52].

	CD	UC	IBD
Age, median (IQR)	40.4 (28.0–62.2)	56.9 (36.0–70.5)	48.0 (30.5–67.0)
Gender			
Female, n (%)	737 (46.9)	649 (52.6)	1386 (49.4)
Male, n (%)	836 (53.1)	585 (47.4)	1421 (50.6)
Age at diagnosis, median (IQR)	36.8 (23.9–58.9)	52.8 (32.3–66.4)	44.2 (27.0–63.3)
BMI at diagnosis, median (IQR)	24.3 (21.1–28.0)	26.0 (22.4–29.6)	25.0 (22.0–29.0)
<18 years, median (IQR)	20.0 (18.0–23.6)	20.0 (17.5–24.2)	20.0 (18.0–24.0)
18–24 years, median (IQR)	22.0 (20.0–25.1)	23.0 (21.0–26.5)	22.3 (20.0-25.9)
25–44 years, median (IQR)	24.4 (22.0–28.5)	25.0 (22.0–29.0)	25.0 (22.0–28.6)
45–64 years, median (IQR)	26.0 (22.2–30.0)	27.0 (24.0–30.2)	26.5 (23.0–30.0)
≥65 years	25.7 (23.0–29.4)	27.0 (23.4–30.2)	26.9 (23.1–30.0)

CD, Crohn’s disease; IBD, inflammatory bowel disease; UC, ulcerative colitis. CD, n = 1573; UC, n = 1234; IBD, n = 2807.

## Abbreviations

APOA4	Apolipoprotein A4
BAT	Brown adipose tissue
BMI	Body mass index
CCK	Cholecystokinin
CCK-1R	CCK_1_ receptor
CCK-2R	CCK_2_ receptor
CD	Crohn’s disease
cAMP	Cyclic adenosine monophosphate
DPP-4	Dipeptidyl peptidase-4
FA	Fatty acid
FOXP3	Forkhead box protein P3
GIP	Glucose-dependent insulinotropic polypeptide
GIP-R	GIP receptor
GLP-1	Glucagon-like peptide-1
GLP-2	Glucagon-like peptide-2
GLP-1R	GLP-1 receptor
HDL	High-density lipoprotein
HFD	High-fat diet
IBD	Inflammatory bowel disease
IFN-γ	Interferon-γ
IL-1α	Interleukin-1α
JNK	c-Jun N-terminal kinase
KO	Knockout
LRP1	Low-density lipoprotein receptor-related protein 1
NF-κB	Nuclear factor kappa B
NPY-Y2	NPY receptor type 2
PI3K	Phosphoinositide 3-kinase
PYY	Peptide YY
SCFA	Short-chain fatty acid
TG	Triglyceride
TNF-α	Tumor necrosis factor-α
UC	Ulcerative colitis
VIP	Vasoactive intestinal polypeptide
WAT	White adipose tissue
